# Inhibition of Type VI Secretion by an Anti-TssM Llama Nanobody

**DOI:** 10.1371/journal.pone.0122187

**Published:** 2015-03-26

**Authors:** Van Son Nguyen, Laureen Logger, Silvia Spinelli, Aline Desmyter, Thi Thu Hang Le, Christine Kellenberger, Badreddine Douzi, Eric Durand, Alain Roussel, Eric Cascales, Christian Cambillau

**Affiliations:** 1 Architecture et Fonction des Macromolécules Biologiques, Centre National de la Recherche Scientifique (CNRS)—UMR 7257, Marseille, France; 2 Architecture et Fonction des Macromolécules Biologiques, Aix-Marseille Université, Campus de Luminy, Case 932, Marseille, France; 3 Laboratoire d’Ingénierie des Systèmes Macromoléculaires, Institut de Microbiologie de la Méditerranée, Aix-Marseille Université, CNRS—UMR 7255, 31 chemin Joseph Aiguier, Marseille, France; Academia Sinica, TAIWAN

## Abstract

The type VI secretion system (T6SS) is a secretion pathway widespread in Gram-negative bacteria that targets toxins in both prokaryotic and eukaryotic cells. Although most T6SSs identified so far are involved in inter-bacterial competition, a few are directly required for full virulence of pathogens. The T6SS comprises 13 core proteins that assemble a large complex structurally and functionally similar to a phage contractile tail structure anchored to the cell envelope by a trans-membrane spanning stator. The central part of this stator, TssM, is a 1129-amino-acid protein anchored in the inner membrane that binds to the TssJ outer membrane lipoprotein. In this study, we have raised camelid antibodies against the purified TssM periplasmic domain. We report the crystal structure of two specific nanobodies that bind to TssM in the nanomolar range. Interestingly, the most potent nanobody, nb25, competes with the TssJ lipoprotein for TssM binding *in vitro* suggesting that TssJ and the nb25 CDR3 loop share the same TssM binding site or causes a steric hindrance preventing TssM-TssJ complex formation. Indeed, periplasmic production of the nanobodies displacing the TssM-TssJ interaction inhibits the T6SS function *in vivo*. This study illustrates the power of nanobodies to specifically target and inhibit bacterial secretion systems.

## Introduction

The type VI secretion system (T6SS) is a machinery widespread in Gram-negative bacteria and dedicated to the delivery of toxins in bacterial and eukaryotic host cells. By its anti-bacterial antagonistic action, the T6SS is one of the main players in the bacterial warfare for the access to nutrients and for colonization of the ecological niche [1, 2]. The T6SS assembles from 13 conserved components. Architecturally, the T6SS can be seen as a micrometer-long syringe anchored to the cell membrane by a trans-envelope complex [3–6]. The phage tail-related syringe-like tubular structure is composed of an internal tube tipped by a spike-like complex, wrapped by a contractile sheath and tethered to the membrane through contacts with components of a trans-envelope multiprotein complex [6, 7]. This membrane-associated complex is composed of the TssL and TssM inner membrane proteins and of the TssJ outer membrane lipoprotein [8–13]. The TssM and TssL proteins interact and stabilize each other and share homologies with the Type IVb secretion system IcmF and DotU subunits respectively [12, 14, 15]. In enteroaggregative *Escherichia coli* (EAEC), TssM (accession number: EC042_4539; gene ID: 387609960) is a 1129-amino-acid protein anchored to the inner membrane by three transmembrane helices and bearing a large ~ 750 amino-acid periplasmic domain (amino-acids 386–1129). The C-terminal extremity of the TssM periplasmic domain interacts with the L1-2 loop of the TssJ lipoprotein with a K_*D*_ of 2–4 μM [11]. By combining interactions with inner membrane and outer membrane-associated components, the TssM protein crosses the cell envelope and is therefore central to the T6SS membrane complex.

Although the EAEC TssM periplasmic domain purified readily, we did not succeed to gain structural information [11]. One of the most efficient approaches to improve the crystallization process is to use co-crystallization of the protein of interest with cognate camelid nanobodies. Camelid (llamas, dromaderies and alpacas) antibodies differ from classical antibodies as they only associate two heavy-chains, lacking the CH1 domain and terminated by monomeric variable antigen-binding V_H_H domains called nanobodies [16, 17, 18]. By contrast to the conventional immunoglobulin domains, these single-domain V_H_H antibodies are highly convenient: in addition to be the smallest antibodies, they are easy to produce in the *E*. *coli* periplasm [19]. Therefore, they have remarkable potential in the biotechnology and bio-pharmaceutical fields [18, 20, 21]. More important for structural biologists, they also demonstrated their efficiency to improve protein solubility and facilitating crystallization when complexed with the protein of interest [19], in particular for membrane-associated or flexible proteins [22, 23, 24, 25, 26]. Finally, due to their high affinity and selectivity and their small size, nanobodies are excellent enzymes and receptors inhibitors and can be used for functional studies.

To gain further information on the EAEC TssM protein, the purified TssM periplasmic domain was used for llama immunization. Here we report the selection and the structural analysis of two specific nanobodies. These antibodies bind to the TssM periplasmic domain with a K_*D*_ in the nanomolar range. One of these nanobodies disrupts the TssM-TssJ interaction *in vitro* and prevents the proper function of the T6SS apparatus.

## Results and Discussion

### Selection and crystal structures of TssM-specific nanobodies

**Fig 1:**
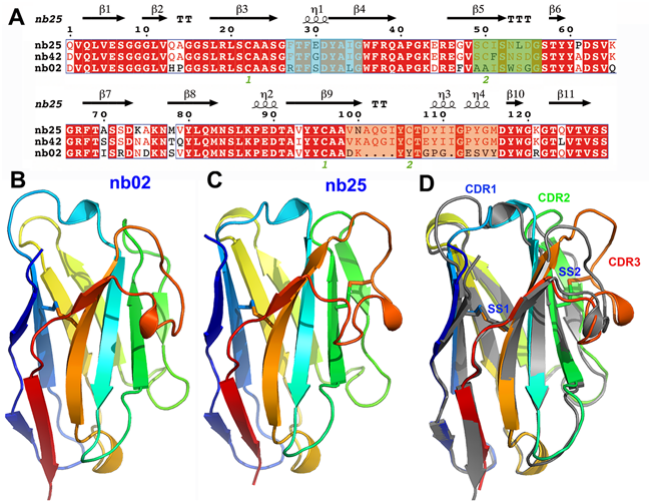
Structure of the anti-TssM nanobodies. (**A**) Sequence alignment of nanobodies nb02, nb25 and nb42. Identical amino-acids are in white and underlined in red, similar amino-acids are colored in red, different amino-acids are in black. The CDR1, CDR2 and CDR3 are highlighted in blue, green and yellow, respectively. The green numbers (1 or 2) below the sequence indicate cysteine residues involved in disulphide bridges SS1 (1, Cys22 and Cys96) and SS2 (2, Cys50 and Cys106) disulphide bridges formation. Crystal structure of nanobodies nb02 (**B**) and nb25 (**C**), represented as ribbons and colored in rainbow mode. (**D**) Superimposition of the structures of nb02 (grey) and nb25 (rainbow color). The locations of the CDR1, CDR2 and CDR3 variable regions are indicated as well as the positions of disulphide bridges (SS1 and SS2).

Nanobodies were raised by immunization of llamas with the purified periplasmic domain of the EAEC TssM protein (TssMp). Three strong TssMp binders were identified from the immune library by three rounds of panning using phage display coupled to ELISA. Two nanobodies, called nb02 and nb25, were selected for further studies based on their high affinity for TssM and on their amino-acid differences in the variable regions, suggesting they bind distinct regions of TssMp (Fig. 1A). The third nanobody, nb42, is very similar to nb25, and was not retained for the structural studies. The two selected nanobodies, nb02 and nb25, sharing 77% sequence identity, were produced in the periplasm of *E*. *coli*, purified to homogeneity and concentrated to 10 mg/ml. Both nb02 and nb25 behaved as monomers in size-exclusion chromatography and both crystallized readily, allowing to solve their three-dimensional structures by molecular replacement (Fig. 1B and 1C). Crystal structures were refined to 1.7 Å and 1. 38 Å resolution with R/Rf values of 25.1/20.5% and 19.1/19.8%, respectively (Table 1). Overall, the two structures (nb02, PDB 4QLR; nb25, PDB 4QGY) are very similar. The superimposed structures of nb02 and nb25 differ by a rmsd of 1.13 Å on 118 aligned residues (Fig. 1D). However, despite identical lengths, the conformations of the nb02 and nb25 CDR1 and CDR2 regions differ significantly (Fig. 1D). More importantly, large differences can be observed on the CDR3 regions, notably in terms of size (14-residue long for nb02, 19-residue long for nb25). Noteworthy, the nb25 CDR3 region is stabilized by a disulfide bridge between Cys-106 and CDR2 Cys-50 and extends out of the nanobody core, providing a large putative binding area for the antigen (Fig. 1C).

**Table 1:** Data collection and refinement statistics for the nb02 and nb25 anti-TssM nanobodies.

DATA COLLECTION	nb02 (PDB:4QLR)	nb25 (PDB:4QGY)
Diffraction source	ESRF	Soleil PX1
Detector	Pilatus 6M	Pilatus 6M
Space group	P 2_1_	C222_1_
Cell dimensions (Å,°)	a = 50.0, b = 48.6, c = 52.9 Å^3^ β = 118.8°	a = 52.0, b = 70.9, c = 145.7 Å^3^
Resolution range^a^ (Å)	50–1.70 (1.76–1.70)	50–1.38 (1.42–1.38)
R-merge^a^ (%)	7.0 (52)	3.1 (70)
CC(1/2)^a^ (%)	99.7 (73.1)	100 (85)
Mean((I)/sd(I))^a^	9.6 (1.7)	29 (2.6)
Total number of reflections^a^	62041 (5333)	395611 (25318)
Number of unique reflections^a^	23496 (2222)	55440 (3886)
Completeness^a^ (%)	94.3 (90.3)	99.6 (95)
Multiplicity^a^	2.64 (2.4)	7.1 (6.5)
**REFINEMENT**		
Resolution^a^ (Å)	44.1–1.7 (1.78–1.7)	20–1.38 (1.42–1.38)
Nr of reflections^a^	23498 (2742)	55416 (3814)
Nr protein / water	1931 / 211	1918 / 309
Nr test set reflections	1201	2813
R_work_/R_free_ ^a^ (%)	20.5/25.1 (24.0/25.4)	19.1/19.8 (32.2/36.7)
r.m.s.d.bonds (Å) / angles (°)	0.01 / 1.02	0.01 / 1.09
B-wilson / B-average	21.0 / 23.6	20.3 / 24.8

^a^ numbers in brackets refer to the highest resolution bin.

### Nanobodies bind TssM with nanomolar affinities

**Fig 2:**
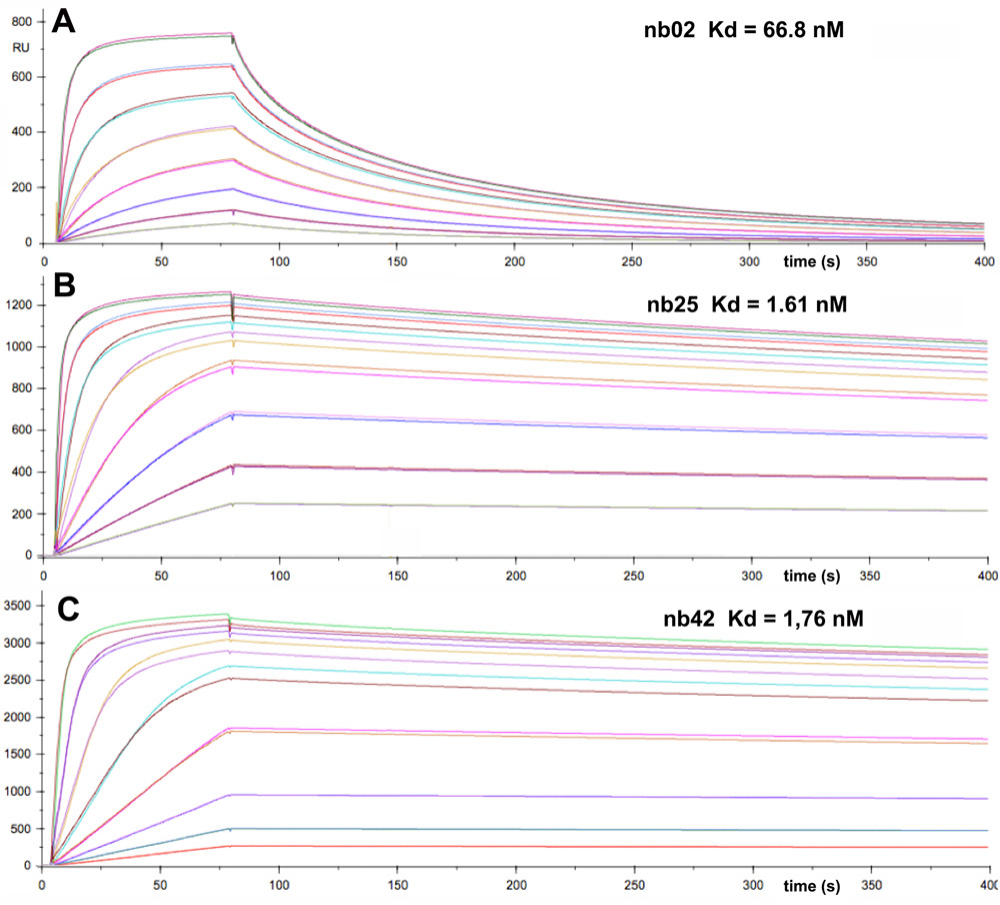
Nanobodies nb02 and nb25 bind TssMp with nanomolar affinity. Surface Plasmon Resonance recordings representing binding and release of the purified periplasmic domain of TssM (from bottom to top: 1.95, 3.9, 7.8, 15.6, 31.25, 62.5, 125 and 250 nM) to nanobody nb02 (**A**), nb25 (**B**) or nb42 (**C**) immobilized on the Chip. The variation of the Surface Plasmon Resonance (shown as the experimental and fitting curves) is reported on the *y* axis (in arbitrary unit, RU) plotted versus the reaction time on the *x* axis (in sec.). The apparent K_*D*_s are indicated on the top of each graph. The kinetic and thermodynamic parameters are indicated in Table 2.

To gain further information on the nanobodies, the strengths of their interactions with TssMp were measured by surface plasmon resonance (SPR). Nb02, nb25 and the nb25 variant nb42 were covalently coupled to the CHIP and sensorgrams were recorded after injection of the purified TssMp fragment in the microfluidic channel (Fig. 2). Analysis of the SPR curves indicate that nb02, nb25 and nb42 bind to TssMp with K_*D*_ values of 66.8±2 nM, 1.61± 0.1 nM and 1.76± 0.1 nM, respectively (Table 2).

**Table 2:** Kinetic and thermodynamic parameters of the interactions between anti-TssM nanobodies with TssMp.

	k_on_ (M^-1^.s^-1^)	k_off_ (s^-1^)	K_D_ (nM)	R_max_
nb02	6.02 10^5^	402 10^-4^	66.8 ± 2.5	734
nb25	3.5 10^5^	5.6 10^-4^	1.61 ± 0.05	1165
nb42	2.04 10^5^	3.6 10^-4^	1.76 ± 0.08	3155

### Nb25 interferes with TssJ binding to TssM

**Fig 3:**
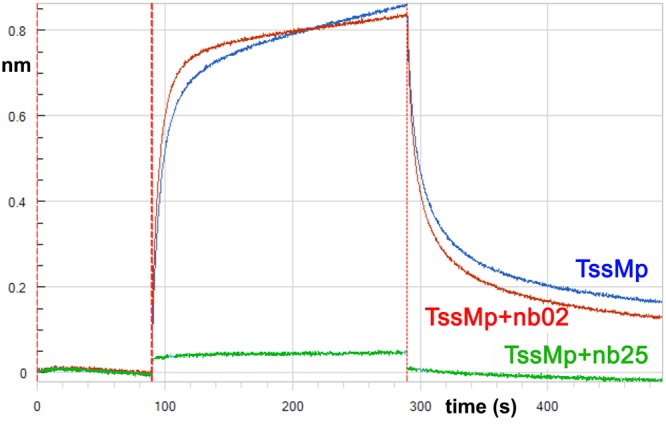
Binding of TssMp:nanobody complexes to TssJ. Bilayer interferometry recordings representing binding of TssMp alone (blue) or the TssMp:nb02 (red) or TssMp:nb25 (green) complexes to chip coupled to TssJ. The response (in nm) is plotted versus the time (in sec.). The absence of response in the green sensorgram indicates that nb25 prevents attachment of TssMp to TssJ.

We previously reported that TssJ binds to TssMp with a K_*D*_ value of 2–4 μM. This interaction is mediated by the L1-2 loop of TssJ and is essential for the proper function of the T6SS [11]. Based on these results, we hypothesized that the TssJ L1-2 loop will contact a crevice within TssM [11]. Because nanobodies are known to target enzymatic sites or crevices [27], we sought to determine whether the nanobodies and TssJ share the same TssM-binding site. We therefore performed Bilayer interferometry (BLI) competition experiments between the nanobodies and TssJ on TssMp. The TssJ protein (devoid of its N-terminal Cys acylation residue) was biotinylated and coupled to the streptavidine BLI chip. The chip was immersed on solution containing TssMp-nanobody complexes. Due to the 50- to 1000-fold magnitude differences between the K_*D*_s of TssJ and that of the nanobodies to TssMp, we expected that the occupation of the TssJ binding site by the nanobody would prevent TssMp binding to TssJ. Fig. 3 shows that the TssMp-nb02 complex can readily interact with TssJ. The formation of the nb02-TssMp-TssJ ternary complex demonstrates that TssJ and nb02 bind TssM differently. By contrast, the TssMp-nb25 complex does not bind TssJ demonstrating that nb25 interferes with TssJ binding on TssMp. This result suggests that nb25 and TssJ share the same TssM binding site or that nb25 binding on TssMp causes steric hindrance preventing TssJ binding.

### Nb25 disrupts the TssMp-TssJ interaction *in vitro*

**Fig 4:**
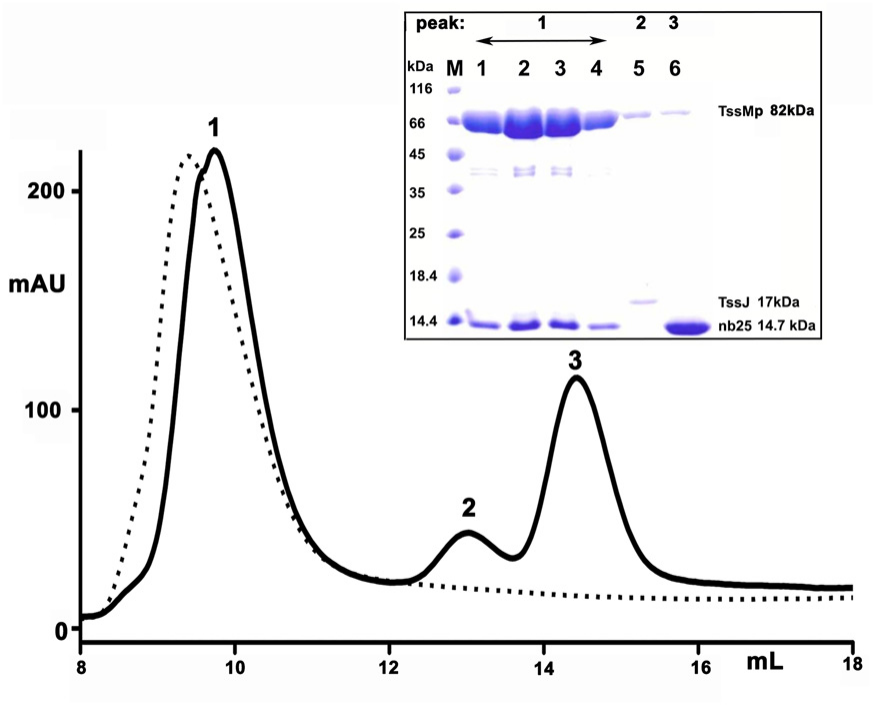
Nanobody nb25 disrupts the TssMp:TssJ complex. The pre-formed TssM-TssJ complex was analyzed by size exclusion chromatography before (dash line) and after incubation with an excess of nb25 (plain line). The composition of the different complexes, eluting at different volumes, was analysed by SDS-PAGE and Coomassie blue staining (inset). The fractions corresponding to peak 1–3 are indicated on the top. The molecular masses of the protein markers (in kDa) are indicated on the left. According to the calibration of the column, the apparent molecular weights for peak 1 (9.72 ml), peak 2 (13.02 ml) and peak 3 (14.43 ml) are about 90, 18 and 14 kDa, respectively. The faint band at the position of TssMp in peaks 2 and 3 of the SDS gel arise from contamination by the large main TssMp peak 1.

We hypothesized that if nb25 and TssJ share the same TssM binding site, nb25 should disrupt the TssM-TssJ complex. We therefore analyzed the effect of nb25 on the stability of the TssM-TssJ complex. Competition experiments were performed by incubating the purified TssMp-TssJ complex with an excess of nb25, prior to analysis by gel filtration. Three peaks were observed on the chromatogram (Fig. 4). Analysis of the peaks by SDS-PAGE (Fig. 4, inset) showed that peak 1 (~ 90 kDa) contained TssMp and nb25 (theoretical weight of the TssMp-nb25 complex = 98 kDa), while peak 2 (~ 18 kDa) contained TssJ (theoretical weight = 17 kDa) and peak 3 (~ 14 kDa), the excess of nb25 (theoretical weight = 14 kDa). These data confirm that nb25 binds to TssMp and chases TssJ from the pre-assembled TssMp-TssJ complex.

### Nb25 prevents formation of a functional T6SS

**Fig 5:**
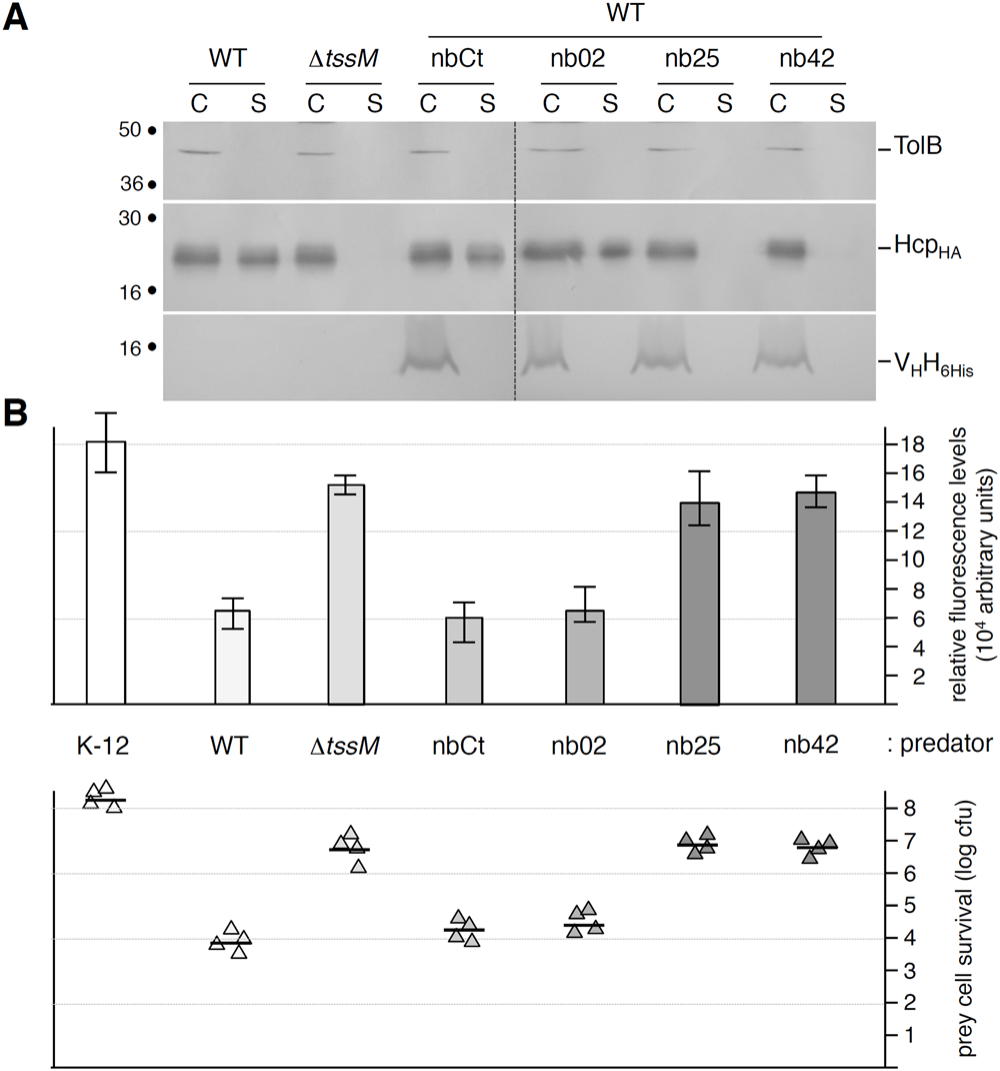
TssM-specific nanobodies nb25 and nb42 specifically affect T6SS function. **(A)** Hcp release assay. HA-epitope-tagged Hcp (Hcp_HA_) release was assessed by separating whole cells (C) and supernatant (S) fractions from wild-type (WT), Δ*tssM c*ells or WT cells producing 6His-tagged nanobodies as indicated. 2×10^8^ cells and the TCA-precipitated material of the supernatant from 5×10^8^ cells were loaded on a 12.5%-acrylamide SDS-PAGE and the nanobodies (lower panel), Hcp (middle panel) and periplasmic TolB (cell integrity control, upper panel) proteins were immunodetected using anti-5His, anti-HA and anti-TolB antibodies respectively. **(B)** Anti-bacterial assay. The Sci-1 T6SS-dependent anti-bacterial activity was assessed by mixing prey cells (W3110 *gfp* ^+^, kan^R^) with the indicated attacker cell (K-12, W3110; WT, EAEC 17–2; Δ*tssM*, 17–2Δ*tssM* or WT cells producing the indicated nanobody) for 16 hours at 37°C in *sci-1*-inducing medium (SIM). The recovered fluorescent level (in arbitrary units) is shown in the upper graph (mean of fluorescence levels per OD_600nm_ obtained from four independent experiments). The number of recovered viable prey cells, expressed in colony forming unit (cfu) is shown in the lower graph (the triangles indicate values from four independent assays, and the average is indicated by the bar).

The TssM-TssJ interaction was previously shown to be indispensable for the proper function of the Type VI secretion apparatus in enteroaggregative *E*. *coli* [11]. As nb25 (and nb42) compete with TssJ for TssM binding *in vitro*, we wondered whether these nanobodies would disrupt the TssM-TssJ interaction *in vivo*. The vectors allowing the periplasmic production of the nb02, nb25 and nb42 nanobodies, as well as a control nanobody (a nanobody targeting the *Lactococcus lactis* phage 1358 receptor binding protein) were introduced into the wild-type EAEC strain 17–2. The function of the T6SS was assessed by the Hcp1 release assay and by the Sci1-dependent antibacterial activity. As shown in Fig. 5, the control nanobody and nb02 had no—or little—effect on the function of the T6SS as 17–2 cells producing nb02 released the Hcp1 protein and retained anti-bacterial activities at levels comparable to that of wild-type 17–2 cells. By contrast, the periplasmic production of nb25 and nb42 inhibited the function of the T6SS as (i) no Hcp1 was found in the culture supernatant of 17–2 cells producing these V_H_Hs and (ii) these cells did not have any growth advantage when co-cultured with *E*. *coli* K-12 cells. Taken together, these results suggest that disruption of the TssM-TssJ interaction prevents the formation of a functional Type VI secretion apparatus in EAEC.

## Concluding Remarks

In this study, we raised and selected two camelid variable fragments of heavy-chain antibodies specific to the EAEC T6SS TssM periplasmic domain. Although this strategy was originally initiated to help the crystallization of the TssM protein, we used here these nanobodies to gain functional information on the assembly of the T6SS apparatus. We showed that the two selected nanobodies bind TssMp with nanomolar affinities. These values are in agreement with the affinities of nanobodies, including that targeting the *Salmonella* Typhimurium actin ADP-ribosylating toxin or the *Pseudomonas aeruginosa* flagellin [28, 29]. BLI and gel filtration experiments showed that nb25 is a competitor of TssJ *in vitro*, suggesting that nb25 binds at—or close to—the TssJ binding site on TssMp or at least causes a steric hindrance or a TssM conformational change preventing TssM-TssJ complex formation. Due to the variability of the CDR3 protruding loop and the observation that nanobodies are often targeting crevices [27], we hypothesize that the nb25 CDR3 loop mediates the interaction with a crevice in TssM, such as the TssJ L1-2 loop does [11]. Significant sequence differences occur in the CDR1 and CDR2 of nb25 and nb42, while the CDR3 are identical. This observation is in line with our hypothesis of CDR3 binding to the TssJ binding site of TssM. Sequence alignment between the TssJ LI-2 loop and the CDR1, CDR2 and CDR3 of nb25 shows that, except an A-X-G-I motif, very limited similarities exist between TssJ and CDR3. However, the TssJ L1-2 loop being elongated whereas the CDR3 having a compact fold, no structural conservation is observed between these loops suggesting that TssJ L1-2 and nb25/nb42 CDR3 do not bind identically to TssM, although we cannot exclude loop rearrangements upon binding. In view of these results, the co-crystallization between TssMp and nb25 is therefore essential, not only to gain structural information on TssM but also to identify the nb25-binding site and to test whether this region is also responsible for making contacts with the TssJ L1-2 loop. Interestingly, the use of nanobodies for co-crystallization was successful in this specific case as we recently obtained co-crystals between a fragment of the periplasmic domain of TssM and nb25 and performed a preliminary X-ray diffraction analysis [30].

By contrast, although nb02 has strong affinity for TssM, we showed that nb02 does not compete with TssJ for TssM binding *in vitro*. Because of the formation of an nb02-TssMp-TssJ ternary complex, nb02 is an interesting tool to assist the crystallization of the TssMp-TssJ complex. Hcp release and growth competition assays showed that nb02 has no inhibitory effect *in vivo*. Therefore, defining the site of binding of nb02 on TssM is also interesting as this nanobody targets a region that is not critical for the assembly of the T6SS membrane complex.

Nanobodies targeting bacterial multi-protein complexes or toxins have already been reported. For examples, specific nanobodies to the *Vibrio* Type II secretion (T2SS) GspD secretin and EspI/J pseudopilin complex have been selected and used for structural studies but their effect on the assembly of the T2SS *in vivo* has not been addressed [31]. By contrast, nanobodies interacting with the *P*. *aeruginosa* or *Campylobacter jejuni* flagellin decrease the swimming rate and biofilm formation of these strains [29, 32]. Similarly, nanobodies that bind to and interfere with the catalytic site of the *S*. Typhimurium ADP-ribosylation toxin diminish the cytotoxicity *in vivo* [28]. Our study not only paves the way to the structural characterization of the TssM periplasmic domain and of the TssMp-TssJ complex, but also demonstrates that nanobodies can be used to inhibit the assembly of the T6SS or of secretion systems in general with the goal to diminish or abolish the fitness or the virulence of bacterial pathogens in their ecological niche.

## Materials and Methods

### Bacterial strains, growth conditions and chemicals

The entero-aggregative *E*. *coli* EAEC strain 17–2 and its Δ*tssM* isogenic derivative [10] were used for this study. The *E*. *coli* K-12 WK6 strain was used for nanobody production. The *E*. *coli* K-12 W3110 strain carrying the pUA66-*rrnB* plasmid (*gfp* under the control of the constitutive *rrnB* ribosomal promoter, specifying strong and constitutive fluorescence, and kanamycin resistance [33]) was used as prey in antibacterial competition experiments. Strains were routinely grown in lysogeny broth (LB) or Terrific broth at 37°C, with aeration. For antibacterial competition assays, cells were grown in *sci-1*-inducing medium (SIM: M9 minimal medium, glycerol 0.2%, vitamin B1 1 μg/ml, casaminoacids 100 μg/ml, LB 10%, supplemented or not with bactoagar 1.5%). Plasmids were maintained by the addition of ampicillin (200 μg/mL) or kanamycin (50 μg/mL). The expression of the nanobody constructs cloned into pHEN6 vector derivatives was induced by the addition of isopropyl-β-thio-galactoside (IPTG).

### Generation of llama nanobodies against TssM

The periplasmic domain of TssM (amino-acids 386–1129; TssMp) was produced and purified as described previously [11]. Four injections of 1 mg of recombinant TssMp (in Tris-HCl 20 mM (pH 8.0), NaCl 150 mM) were performed subcutaneously with two weeks intervals followed by a fifth injection one month later in two llamas (*Lama glama*) (Capralogics Inc., Hardwick, MA 01037 USA). Lymphocytes were isolated from blood sample obtained 1 week after the last immunization. cDNA was synthesized from purified total RNA by reverse transcription. The cDNA was used as template for PCR amplification to amplify sequences corresponding to the variables domains of the heavy-chain antibodies. PCR fragments were cloned into the phagemid vector pHEN4 [34] to create a nanobody phage display library. Selection and screening of nanobodies were performed as previously published [35]. Three rounds of panning resulted in the isolation of TssMp-specific binders. Nanobodies nb02, nb25 and its variant nb42 were selected, sequenced and cloned into the pHEN6 expression vector downstream the *pelB* signal peptide and fused to a C-terminal 6×His tag [36].

### Purification of nanobodies

*E*. *coli* WK6 cells carrying the pHEN6 derivatives were grown at 37°C in Terrific Broth medium containing 0.1% glucose and ampicillin to an optical density (OD_600nm_) ~ 0.8. The expression of the nanobody was induced by the addition of 1 mM IPTG and incubation for 16 hours at 28°C. The periplasmic fraction containing the nanobodies was prepared by osmotic shock. The His-tail-containing fusion proteins were purified by immobilized metal affinity chromatography on a 5-mL Ni-NTA column equilibrated in 50 mM Na/K phosphate (pH 8.0), 300 mM NaCl, 10% glycerol. Nanobodies were eluted in 250mM imidazole and concentrated (Amicon-Ultra 10-kDa cut-off) prior to be loaded on a HiLoad 16/60 Superdex 75 gel filtration column equilibrated in 20 mM Tris–HCl (pH 8.0), 150 mM NaCl.

### Nanobodies structure determination

Crystallization screening experiments were performed with several commercial kits. The nano-drop crystallization experiments were performed in Greiner plates. The reservoirs of the Greiner plates were filled up using a TECAN pipetting robot, while the nano-L drops were dispensed by a Mosquito robot using nano-crystallization protocols [37]. All crystallization experiments were performed at 293K. Nanobody nb02 crystallized at 15 mg/mL in 100 mM sodium cacodylate (pH 6.5), 25% w/v PEG 8000, 200 mM (NH_4_)_2_SO_4_. Nanobody nb25 crystallized at 15 mg/mL in 100 mM CHES (pH 10), 1 M K/Na tartrate, 200 mM Li_2_SO_4_. Crystals were mounted in loops and soaked in their crystallization solution supplemented by 4M TMAO before cryocooling.

Data collection of nb02 and nb25 were performed at synchrotrons ESRF (Grenoble, France) beamline ID29 and Soleil PX1 (saint-Aubin, France), respectively (Table 1). Data were integrated with XDSME and scaled with XSCALE. The nb02 crystals belong to space-group P2_1_, with cell dimensions a = 50.0, b = 48.6, c = 52.9 Å^3^, β = 118.8°. Two molecules in the symmetric unit yield a Vm value of 2.0 Å^3^/Da and 38% solvent. The nb25 crystals belong to space-group C222_1_, with cell dimensions a = 52.0, b = 70. 9, c = 145.7 Å^3^. Two molecules in the asymmetric unit yielded a Vm value of 2.20 Å^3^/Da and 45% solvent. These crystals diffracted to 1.70 Å and 1.38 Å resolution, respectively (Table 1). Molecular replacement was performed using MOLREP [38] and nanobody structures with high sequence similarity to nb25 (PDB: 4KRP) and nb02 (PDB: 4HEP) as starting models. Refinement was performed using autoBUSTER [39] alternated with rebuilding with COOT [40].

### Accession numbers

Atomic coordinates have been deposited in the Protein Data Bank as identifiers 4QLR (nb02) and 4QGY (nb25).

### Surface Plasmon Resonance (SPR) measurements

The interaction between TssMp and nanobodies was tested by SPR using a Biacore X100 (GE healthcare). Nb02, nb25 and nb42 were covalently linked to a CM5 chip and different concentrations of TssMp were injected in the microfluidic channel. Regeneration was achieved by the injection of glycine-HCl (pH 2.5) buffer. The K_*D*_ values were calculated by the multi-cycle kinetics method (Biacore, GE-healthcare).

### Biolayer interferometry (BLI)

TssJ was first biotinylated using the EZ-Link NHS-PEG4-Biotin kit (Perbio Science, France). The reaction was stopped by removing the excess of the biotin using a Zeba Spin Desalting column (Perbio Science, France). BLI studies were performed in black 96-well plates (Greiner) at 25°C using an OctetRed96 (ForteBio, USA). Streptavidin biosensor tips (ForteBio, USA) were first hydrated with 0.2 ml Kinetic buffer (KB, ForteBio, USA) for 20 min and then loaded with biotinylated TssJ (10 μg/ml in KB). The association of TssJ with TssM (300 nM) was monitored for 200 sec, as well as the dissociation in KB. For TssM/nanobody competition experiments, nb02 or nb05 nanobodies were used at a TssJ:nanobody ratio of 3:1.

### Size Exclusion Chromatography (SEC)

Size exclusion chromatography was performed on an Alliance 2695 HPLC system (Waters) using a pre-calibrated Superdex 75 10/300 GL gel filtration column run in 20 mM Tris-HCl (pH 8.0), 100 mM NaCl at 0.5 mL/min.

### Hcp release assay

The expression of the nanobody constructs cloned into pHEN6 vector derivatives was induced by the addition of 0.5 mM isopropyl-β-thio-galactoside (IPTG) for 45 min. Supernatant and cell fractions were separated as previously described [12, 13] with the addition of the iron chelator 2,2’-dipyridyl (125 μM final concentration) to the culture medium 30 min before harvesting the cells to induce the expression of the *sci-1* T6SS [41]. Briefly, 2 × 10^9^ cells producing HA epitope-tagged Hcp (from plasmid pOK-HcpHA; [10]) and the 6×His-tagged nanobody constructs were harvested and collected by centrifugation at 2,000 × *g* for 5 min. The supernatant fraction was then subjected to a second low-speed centrifugation and then at 16,000 × *g* for 15 min. The supernatant was filtered on sterile polyester membranes with a pore size of 0.2 μm (membrex 25 PET, membraPure GmbH) before overnight precipitation with trichloroacetic acid (TCA) 15% on ice. Cells and precipitated supernatants were resuspended in loading buffer and analyzed by Sodium Dodecyl Sulfate-Polyacrylamide Gel Electrophoresis (SDS-PAGE) and immunoblotting with the anti-HA antibody. As control for cell lysis, Western blots were probed with antibodies raised against the periplasmic TolB protein. The production of the nanobodies was verified by immunoblotting with the anti-5His antibody.

### Sci-1-dependent antibacterial assay

The antibacterial competition growth assay was performed as described for the studies on the *Citrobacter rodentium* and EAEC Sci-2 T6SSs [42, 43] with modifications. The wild-type *E*. *coli* strain W3110 bearing the pUA66-*rrnB* plasmid Kan^R^ [33] was used as prey in the competition assay. The pUA66-*rrnB* plasmid provides a strong constitutive green fluorescent (GFP^+^) phenotype. Attacker and prey cells were grown for 16 hours in LB medium, then diluted 100-fold in SIM. Once the culture reached an OD_600nm_ = 0.8, the cells were harvested and resuspended to an OD_600nm_ of 0.5 into SIM. For the attacker cells, the expression of the V_H_H-encoding genes was induced for 45min. with 0.5 mM IPTG prior to cell harvest. Attacker and prey cells were mixed to a 4:1 ratio and 25-μl drops of the mixture were spotted in triplicate onto a pre-warmed dry SIM agar plate supplemented with 0.5 mM IPTG. After overnight incubation at 30°C, the bacterial spots were cut off, and cells were resuspended in LB to an OD_600nm_ of 0.5. Triplicates of 150 μl were transferred into wells of a black 96-well plate (Greiner), and the absorbance at 600 nm and fluorescence (excitation, 485 nm; emission, 530 nm) were measured with a Tecan Infinite M200 microplate reader. The relative fluorescence was expressed as the intensity of fluorescence divided by the absorbance at 600 nm, after subtracting the values of a blank sample. For enumeration of viable prey cells, bacterial suspensions recovered from the spots were serially diluted and spotted onto selective LB agar plates supplemented with kanamycin (for the *E*. *coli* prey cells).

### Miscellaneous

SDS-Polyacrylamide gel electrophoresis was performed using standard protocols. For immunostaining, proteins were transferred onto nitrocellulose membranes, and immunoblots were probed with primary antibodies, and goat secondary antibodies coupled to alkaline phosphatase, and developed in alkaline buffer in presence of 5-bromo-4-chloro-3-indolylphosphate and nitroblue tetrazolium. The anti-TolB polyclonal antibodies are from our laboratory collection, while the anti-HA (3F10 clone, Roche), the anti-5His (Qiagen) monoclonal antibodies and alkaline phosphatase-conjugated goat anti-rabbit, mouse, or rat secondary antibodies (Millipore) have been purchased as indicated.

### Ethics statement

Please note that Eric Cascales is a PLOS ONE Academic Editor. This does not alter the authors’ adherence to all the PLOS ONE policies on sharing data and material.
